# The Adaptor Protein Myd88 Is a Key Signaling Molecule in the Pathogenesis of Irinotecan-Induced Intestinal Mucositis

**DOI:** 10.1371/journal.pone.0139985

**Published:** 2015-10-06

**Authors:** Deysi V. T. Wong, Roberto C. P. Lima-Júnior, Cibele B. M. Carvalho, Vanessa F. Borges, Carlos W. S. Wanderley, Amanda X. C. Bem, Caio A. V. G. Leite, Maraiza A. Teixeira, Gabriela L. P. Batista, Rangel L. Silva, Thiago M. Cunha, Gerly A. C. Brito, Paulo R. C. Almeida, Fernando Q. Cunha, Ronaldo A. Ribeiro

**Affiliations:** 1 Nucleus for the Study of Toxicities of the Cancer Treatment, Department of Physiology and Pharmacology, Faculty of Medicine–Federal University of Ceará, Fortaleza, Brazil; 2 Laboratory of Molecular Biology, Department of Pathology, Cancer Institute of Ceará, Fortaleza, Brazil; 3 Department of Pathology and Forensic Medicine, Faculty of Medicine–Federal University of Ceará, Fortaleza, Brazil; 4 Department of Pharmacology, School of Medicine of Ribeirão Preto, University of São Paulo, São Paulo, Brazil; 5 Department of Morphology, Faculty of Medicine–Federal University of Ceará, Fortaleza, Brazil; 6 Department of Clinical Oncology, Cancer Institute of Ceará, Fortaleza, Brazil; Centre d'Immunologie de Marseille-Luminy, CNRS-Inserm, FRANCE

## Abstract

Intestinal mucositis is a common side effect of irinotecan-based anticancer regimens. Mucositis causes cell damage, bacterial/endotoxin translocation and production of cytokines including IL–1 and IL–18. These molecules and toll-like receptors (TLRs) activate a common signaling pathway that involves the Myeloid Differentiation adaptor protein, MyD88, whose role in intestinal mucositis is unknown. Then, we evaluated the involvement of TLRs and MyD88 in the pathogenesis of irinotecan-induced intestinal mucositis. MyD88-, TLR2- or TLR9-knockout mice and C57BL/6 (WT) mice were given either saline or irinotecan (75 mg/kg, i.p. for 4 days). On day 7, animal survival, diarrhea and bacteremia were assessed, and following euthanasia, samples of the ileum were obtained for morphometric analysis, myeloperoxidase (MPO) assay and measurement of pro-inflammatory markers. Irinotecan reduced the animal survival (50%) and induced a pronounced diarrhea, increased bacteremia, neutrophil accumulation in the intestinal tissue, intestinal damage and more than twofold increased expression of MyD88 (200%), TLR9 (400%), TRAF6 (236%), IL–1β (405%), IL–18 (365%), COX–2 (2,777%) and NF-κB (245%) in the WT animals when compared with saline-injected group (*P*<0.05). Genetic deletion of MyD88, TLR2 or TLR9 effectively controlled the signs of intestinal injury when compared with irinotecan-administered WT controls (*P*<0.05). In contrast to the MyD88^-/-^ and TLR2^-/-^ mice, the irinotecan-injected TLR9^-/-^ mice showed a reduced survival, a marked diarrhea and an enhanced expression of IL–18 versus irinotecan-injected WT controls. Additionally, the expression of MyD88 was reduced in the TLR2^-/-^ or TLR9^-/-^ mice. This study shows a critical role of the MyD88-mediated TLR2 and TLR9 signaling in the pathogenesis of irinotecan-induced intestinal mucositis.

## Introduction

Many anti-tumor agents used to treat cancer also affect rapidly dividing normal cells, including the epithelial cells of the gut, leading to various degrees of mucositis in the gastrointestinal tract [**1**]. Of particular importance in the clinical setting is the life-threatening intestinal mucositis associated with diarrhea [**1**]. The use of chemotherapeutic protocols that include irinotecan may cause diarrhea in as many as 80% of patients and in 5–47% of them, a severe form of diarrhea (National Cancer Institute–Common Toxicity Criteria grades 3 or 4) is reported [**1–3**]. The clinical consequences of chemotherapy-induced diarrhea include dose-reduction (45%), delays in therapy (71%) and discontinuation of therapy (3%) [**1**].

Irinotecan, a topoisomerase I inhibitor, is clinically active against lung, gastric, cervical and ovarian cancers and is used as first- and second line-therapy for colorectal cancer [**4**]. Irinotecan-related diarrhea occurs in two forms, an early-onset type, which is dependent on the activation of cholinergic signaling and is controlled with atropine, and a late-onset diarrhea, whose mechanism has not been completely elucidated. However, loperamide, a μ_2_-opioid agonist agent used as a first-line treatment, and octreotide, whose mechanism is unclear and is used as a second-line treatment, are somewhat effective [**1**]. Recently, we demonstrated that in spite of regulating the diarrheic events, the inflammatory response associated with the mucositis induced by irinotecan is not attenuated by loperamide [**5**], which might explain its incomplete response in clinical setting. Other and less effective prophylactic approaches that have been suggested to manage chemotherapy-induced diarrhea include octreotide, glutamine, celecoxib, activated charcoal, absorbents, and racecadotril [**6**]. However, an improved knowledge concerning the pathogenesis of mucositis would open substantial perspectives on the adequate management of this condition.

The intestinal microbiota is believed to influence all phases of the pathogenesis of mucositis [**7**]. Activation of the innate immune response in the intestine is triggered by pathogen-recognition receptors including toll-like receptors (TLRs), which function as sensors of pathogen-associated molecular patterns (PAMPs) and damage-associated molecular patterns (DAMPs) [**8**].

TLRs are transmembrane proteins that maintain intestinal homeostasis [**9**] or contribute to inflammatory diseases [**10**]. TLRs initiate the innate immune response and the production of pro-inflammatory mediators including interleukin–1β (IL–1β) [**11**], nitric oxide [**12**] and interleukin–18 [**13**], whose role in intestinal mucositis has been previously described by our group [**5,14,15**]. Several TLRs and the IL–1 family of cytokines are known to signal through a complex intracellular network that involves MyD88-dependent or Myd88-independent pathways [**16**] that regulate the mucosal immune response [**17,18**]. Interestingly, Frank and colleagues [**19**] showed that TLR2 knockout mice injected with methotrexate present an exacerbated mucositis. In addition, Sukhotnik et al [**20**] suggested that MyD88-dependent TLR4 signaling is protective against methotrexate-induced intestinal damage. In contrast, Kaczmarek and co-workers [**21**] demonstrated that TLR2 and TLR9 signaling pathways play a central role in the development of doxorubicin-induced intestinal mucositis. However, in contrast to irinotecan, protocols that include doxorubicin are mainly described to induce severe oral mucositis (up to 13.40% of patients) rather than intestinal damage and severe diarrhea (2.78% of patients) [**22**]. Such findings might suggest that the pathogenesis of mucositis seems to be strongly dependent on the chemotherapeutic agent used

Then, we aimed to study the involvement of TLR2, TLR9 and the downstream adaptor molecule, MyD88, in the pathogenesis of irinotecan-induced intestinal.

## Materials and Methods

### Animals

The experiments were performed on male C57BL/6 mice (background mouse strain, wild type, WT, 20–24 g) and MyD88 (MyD88^-/-^)-, Toll-like receptor 2 (TLR2^-/-^)- and 9 (TLR9^-/-^)-deficient mice. The mice were housed in the animal care facility of the School of Medicine of Ribeirão Preto and were divided in experimental groups of 6–9 animals. The animals were kept in a temperature-controlled room under a dark-light cycle, and food and water were available *ad libitum*. The original breeding pairs of mice with the targeted disruptions of the TLRs and MyD88 gene were obtained from The Jackson Laboratories (Bar Harbor, Maine, USA). The genotype of these mice was confirmed by DNA PCR.

### Ethics statement

This study was carried out in strict accordance with the recommendations in the Guide for the Care and Use of Laboratory Animals of the National Institutes of Health and the ARRIVE Guidelines (Animals in Research: Reporting In Vivo Experiments) [**23**]. All efforts were made in order to minimize animal suffering. In the survival study, the animals were monitored twice daily for ten days following the first injection of irinotecan. During the experiment, fifty percent of the animals succumb due to the treatment and its consequences, including diarrhea. Those animals that showed signs of imminent death, including piloerection, reduced locomotion, inability to maintain upright position, ataxia, tremor and altered breath frequency were euthanized by ketamine/xylazine overdose (>100/10 mg/kg, s.c., União Química, São Paulo, Brazil) followed by cervical dislocation. Pain relievers or anesthesia were not used in our experiments since those agents directly interfere with the production of inflammatory mediators and/or alter the gastrointestinal transit and mask the diarrheic events in this animal model. At the end of the survival experiment, live animals were euthanized by ketamine/xylazine overdose (>100/10 mg/kg, s.c., União Química, São Paulo, Brazil) followed by cervical dislocation. The experimental protocol, including the mortality aspects of the protocol, was reviewed and approved by the Committee on the Ethics of Animal Experiments of the Federal University of Ceará (Permit Number: 99/10).

### Drugs

Irinotecan hydrochloride (irinotecan, Evoterin®, Evolabis, São Paulo, Brazil, 100 mg ampoule) and sterile saline were used.

### Induction of experimental intestinal mucositis

The induction of experimental intestinal mucositis in mice was based on a model previously described by Ikuno et al. [**24**], and modified for our experimental conditions. Briefly, C57BL/6 wild type, MyD88^-/-^, TLR2^-/-^ and TLR9^-/-^ mice were given either saline (3.5 mL/kg, i.p.) or irinotecan (75 mg/kg, i.p.) once daily for 4 days. On day seven after the first dose of irinotecan diarrhea, and blood leukocyte and bacterial counts were assessed. The animals were anesthetized with an overdose of ketamine/xylazine (>100/10 mg/kg, s.c.) followed by cervical dislocation for the collection of samples of the ileum for morphometric analysis, myeloperoxidase assay, NF-κB immunohistochemistry, Interleukin–1 levels and COX–2 and IL–18 mRNA expression. For the study of animal survival, the mice were observed as described previously up to day ten following the first injection of irinotecan.

### Diarrhea assessment

Diarrhea observed on the seventh day after the first dose of irinotecan was considered to be delayed-onset diarrhea. The severity of the diarrhea was scored as described by Kurita et al. [**25**] as follows: 0 (normal), normal stool or absent; 1 (slight), slightly wet and soft stool; 2 (moderate), wet and unformed stool with moderate perianal staining of the coat; and 3 (severe): watery stool with severe perianal staining of the coat.

### Blood leukocyte and bacterial counts

The mice were lightly anaesthetized with tribromoethanol 2.5% solution (10 mL.kg^−1^, i.p.), and a sample of blood was collected from the retro-orbital plexus. The blood leukocyte count was performed using a Coulter ACT series cell counter and expressed as cells x 10^3^.μL^−1^ of blood. A bacterial count in whole blood was conducted as previously described by Godshall et al. [**26**]. Briefly, blood samples were collected under sterile conditions, plated in Muller-Hinton agar dishes (Difco Laboratories), and aerobically incubated at 37°C. Colony-forming units (CFU) were recorded after 48 h of culture. The results were expressed as the log of CFU.mL^−1^ of blood.

### Morphometric analysis

The specimens were fixed in 10% neutral buffered formalin, dehydrated, and embedded in paraffin. Sections of 5 μm thickness were obtained for hematoxylin-eosin staining (H&E) and subsequent examination by light microscopy (x100). For the morphometric analysis, the length of the intestinal villi was measured using Software ImageJ 1.4 (NIH–National Institute of Health, Bethesda, MD, USA). Between 5 and 10 villi were measured per slice [**5**]. Mucosal injury was also assessed using a modification of the histopathological score system described by Macpherson & Pfeiffer [**27**] and was graded as follows: **Score 0**, normal histological findings; **Score 1**, mucosa: villus blunting, loss of crypt architecture, sparse inflammatory cell infiltration, vacuolization and oedema. Muscle layer: normal. **Score 2**, mucosa: villus blunting with fattened and vacuolated cells, crypt necrosis, intense inflammatory cell infiltration, vacuolization and oedema. Muscle layer: normal. **Score 3**, mucosa: villus blunting with fattened and vacuolated cells, crypt necrosis, intense inflammatory cell infiltration, vacuolization and oedema. Muscle layer: oedema, vacuolization and neutrophilic infiltration.

### Determination of ileum tissue myeloperoxidase activity (MPO)

MPO is an abundant enzyme found in the azurophilic granules of neutrophils, and the measurement of myeloperoxidase is commonly used as a neutrophil marker in inflamed tissue as previously described [**28**]. Briefly, a sample of the ileum (40–60 mg) was harvested and homogenized in 0.02 M NaPO_4_ buffer (pH 4.7) containing 0.1 M NaCl and 0.015 M Na_2_ EDTA then centrifuged at 800 g for 15 min at 4°C. The pellet was then subjected to hypotonic lysis (0.2% NaCl solution, followed 30 s later by the addition of an equal volume of a solution). After a further centrifugation step, the pellet was resuspended in 300 μL of 0.05 M NaPO_4_ buffer, pH 5.4, containing 0.5% hexadecyltrimethyl-ammonium bromide (HTAB, Sigma). The MPO activity was developed with the color reagent tetramethylbenzidine (1.6 mM) and H_2_O_2_ (0.5 mM). The reaction was stopped with a 2 M H_2_SO_4_ solution and the absorbance at 450 nm was determined using a spectrophotometer. The readings were compared with those of a standard curve of mouse peritoneal neutrophils processed in the same way, and the data obtained were expressed as MPO activity (neutrophils/mg of tissue).

### NF-κB immunohistochemistry

Immunohistochemistry for the NF-κB p50 nuclear localization sequence (NLS) was performed using the streptavidin-biotin-peroxidase method [**29**]. Ileal cross-sections were processed and incubated overnight (4°C) with primary rabbit anti-NF-κB antibody (Santa Cruz Biotechnology, sc–114) diluted 1:400 in PBS with bovine serum albumin (PBS-BSA). The slides were then incubated with biotinylated goat anti-rabbit antibody (Santa Cruz Biotechnology) diluted 1:800 in PBS/BSA. After washing, the slides were incubated with the avidin-biotin-horseradish peroxidase conjugate (Strep ABC complex by Vectastain^®^ ABC Reagent and peroxidase substrate solution) for 30 min, according to the Vectastain protocol. NF-κB was visualized with the chromogen 3,3’-diaminobenzidine (DAB). The negative control sections were processed simultaneously as described above except that the first antibody was replaced with PBS-BSA 5%. Qualitative immunohistochemistry was performed as described by [**30**]. The staining was observed using light microscopy. NF-κB expression was evaluated by counting the immunostained nuclei in the Lieberkühn crypts and expressed as the percentage of positive stained nuclei.

### Quantification of nuclear p65 subunit as a parameter of NF-κB activation by western blotting

Ileum samples of WT or Myd88^-/-^ mice were lysed, homogenized and immediately transferred to tubes and vortexed for 1 min. The resulting extract was centrifuged at 10,000 g for 5 min and the supernatant was collected as a cytoplasmic extract. The pellet was washed and following centrifugation the supernatant was discarded. The resulting pellet was lysed, maintained in ice and homogenized by vortexing for 30 min. The resulting extract was centrifuged at 10,000 g for 5 min. The resulting supernatant was taken as nuclear extract. The proteins in each extract were separated by electrophoresis on 12% polyacrylamide gel (SDS-PAGE) followed by transfer to nitrocellulose membrane. The membrane containing the nuclear proteins was incubated with 1:300 rabbit anti-p65 (sc–372, Santa Cruz Biotechnology, Dallas, TX, USA), washed and incubated with a secondary antibody conjugated with peroxidase (anti-rabbit IgG, Sigma, St. Louis, MO, USA). For measurement, the chemiluminescence system was visualized using the ChemiDoc^TM^ XRS+ System (BioRad, Life Technologies, Carlsbad, CA, USA). To assess the quality of the separation of the extracts, the same membranes were stained with the anti-mouse antibody nucleophosmin (Sigma, St. Louis, MO, USA). Then, the membranes were incubated with respective secondary antibodies conjugated with peroxidase following the same protocol described above. The bands shown are representative of the groups. The quantification was performed by normalization with control group (medium) [**31**].

### IL–1β enzyme-linked immunosorbent assay (ELISA)

Intestinal samples of MyD88^-/-^, TLR2^-/-^ and TLR9^-/-^ mice were removed and processed as described by Safieh-Garabedian et al. [**32**] to determine the concentration of interleukin (IL)-1β using an enzyme-linked immunosorbent assay (ELISA) [**33**]. Briefly, microtiter plates were coated overnight at 4°C with an antibody against mouse IL–1β (4 μg/mL, DuoSet ELISA Development kit R&D Systems). After blocking the plates, the sample and standard were added and incubated at 4°C for 2 h. After washing the plates, biotinylated goat anti-mouse (diluted 1:1000 with assay buffer 1% BSA, R&D System, USA) was added to the wells at room temperature for 2 h and incubated with 100 μl of Streptavidin-HRP diluted 1:200. The substrate solution (100 μL of a 1:1 mixture of H_2_O_2_ and tetramethylbenzidine; R&D System, USA) was then added to the plate, and incubated in the dark at room temperature for 20 min. The enzyme reaction was stopped with H_2_SO_4_ (2N) and absorbance was measured at 450 nm. The results are expressed as pg/mg of tissue and reported as the means ± S.E.M.

### Quantitative real-time polymerase chain reaction (qRT-PCR)

Ileum samples from the WT mice were removed to determine the expression of MyD88, *Tlr2*, *Tlr9*, *Myd88* and *Traf6*. In addition, intestinal samples of MyD88^-/-^, TLR2^-/-^ and TLR9^-/-^ mice were removed to quantify the expression of cyclooxygenase–2 (*Cox–2*) and *IL–18*. Total RNA isolation was performed using the *Aurum* ^*TM*^ *Total RNA Fatty and Fibrous Tissue Kit* (Bio-Rad, CA, USA). The yield and quality of total RNA were determined spectrophotometrically using 260 nm and a 260/280-nm ratio, respectively. One microgram of total RNA from the intestinal samples in a final volume of 20 μl were reverse-transcribed into cDNA in the C1000 Touch^TM^ Termal Cycler system with the iScript^TM^ cDNA synthesis kit from Bio-Rad. Real-time quantitative PCR analysis of the mRNA was performed in an CFX96 Touch^TM^ real-time PCR detection system instrument from Bio-Rad using the iQTM SYBR® Green Supermix (Bio-Rad, CA, USA) as indicated by the manufacturer. All samples were run in duplicate, and the relative mRNA expression level was determined after normalizing all values to those of β-actin. All samples were evaluated for the dissociation characteristics of the double-stranded DNA during heating (melting curve analysis). The relative gene expression was determined using the 2^-ΔΔCt^ method [**34**] with β-actin as the housekeeping gene. The primer pairs used in this study are shown in **Table 1**.

**Table 1:** Primers used in this study.

Primers		Sequence
***Myd88***	Forward	5'- GCCTTTACAGGTGGCCAGAG–3'
	Reverse	5´- CGGATCATCTCCTGCACAAA–3'
***Tlr2***	Forward	5'-CAAACGCTGTTCTGCTCAGG–3'
	Reverse	5´- CACCATGGCCAATGTAGGTG–3’
***Tlr9***	Forward	5’- TCCATCACCTGAGCCATCTG–3’
	Reverse	5’- TAGGTCCAGCACCGAGAGGT–3’
***Traf6***	Forward	5’-AGCGCTGCAGTGAAAGATGA–3’
	Reverse	5’-CTGCTTCCCGTAAAGCCATC–3’
***Cox–2***	Forward	5'-GTGGAAAAACCTCGTCCAGA–3'
	Reverse	5´-GCTCGGCTTCCAGTAFFGAG–3'
***Il–18***	Forward	5'-CAGGCCTGACATCTTCTGCAA–3’
	Reverse	5´- TCTGACATGGCAGCCATTGT–3’
***β-actin***	Forward	5′-GACATGGAGAAGATCTGGCA–3′
	Reverse	5′-GGTCTTTACGGATGTCAACG–3′

### Statistical analysis

The parametric data are expressed as the means ± standard error of the mean (S.E.M.), except for the diarrhea assessment and histopathologic scores (non-parametric data), which are reported as the median values (minimum-maximum). The data were analyzed using one-way or two-way ANOVA followed by Bonferroni’s test (parametric data) or by Kruskal-Wallis followed by Dunn’s test (non-parametric data). The Mantel-Cox log rank test was used to determine differences between survival curves. Statistical significance was accepted when *P<*0.05.

## Results

### TLR and downstream molecules are expressed during intestinal mucositis

**Fig 1:**
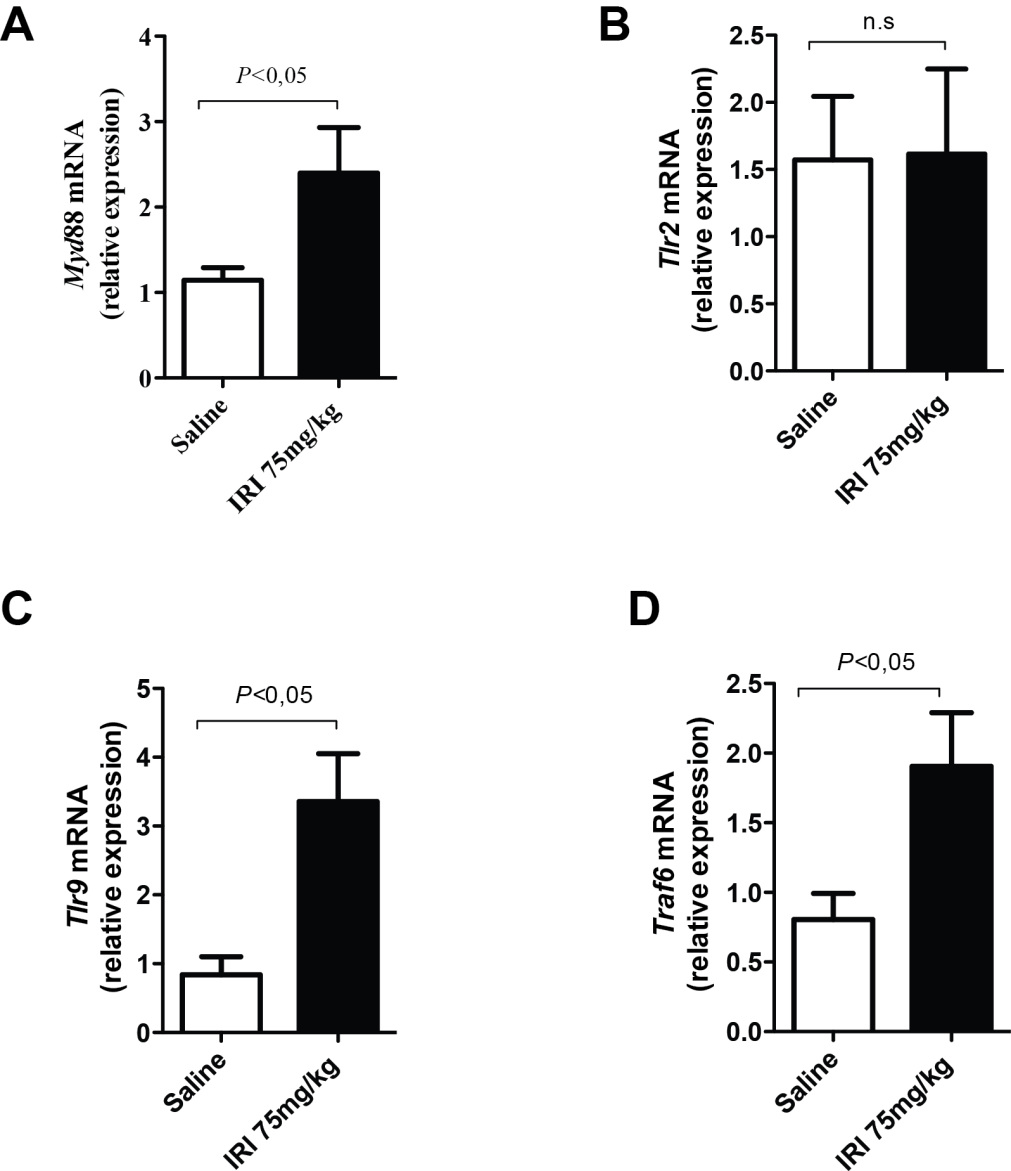
TLR9, MyD88 and TRAF6 are markedly expressed during irinotecan-induced intestinal mucositis. The mice (n = 6–9) were injected for 4 days with saline (3 mL/kg) or irinotecan (75 mg/kg, i.p.) and were killed on the seventh day after the first dose. Ileal samples were collected and processed for quantitative PCR. The expression of MyD88 (panel A), TLR9 (panel C) and TRAF6 (panel D), but not of TLR2 (panel B), was markedly increased in irinotecan-injected WT mice when compared with saline-injected animals. The values are expressed as the means ± SEM.

As shown in **Fig 1**, mRNA expression of MyD88 (twofold increase, panel A), TLR9 (fourfold increase, panel C) and TRAF6 (twofold increase, panel D), but not of TLR2 (panel B), are significantly increased in WT mice that received irinotecan in comparison with saline-injected group (P<0.05).

### MyD88-, TLR2- or TLR9- knockout mice show a preserved intestinal architecture during intestinal mucositis

**Fig 2:**
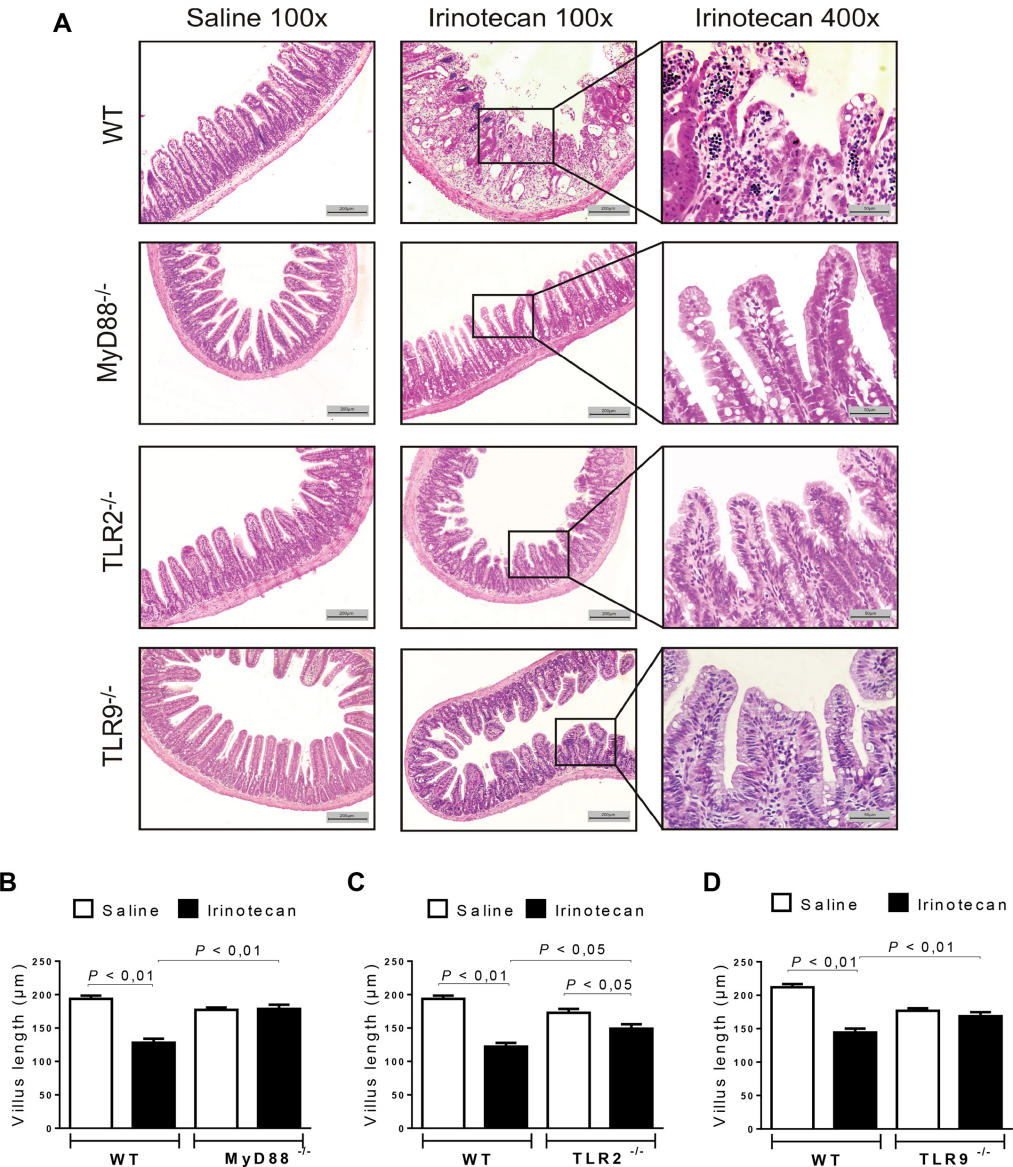
Irinotecan-induced intestinal injury is attenuated in MyD88^-/-^, TLR2^-/-^ and TLR9^-/-^ mice. The mice (n = 6–9) were injected for 4 days with saline (3 mL/kg) or irinotecan (75 mg/kg, i.p.) and were killed on the seventh day after the first dose. Ileal samples were collected and processed for histopathological and morphometric analysis. The irinotecan-treated WT animals showed a pronounced intestinal injury and inflammatory infiltration (**A**) and shorter villi lengths (**B-D**) versus the saline-treated WT group (**A-D**). The MyD88-, TLR2- and TLR9- knockout mice demonstrated a more intact gut architecture (**A**) and increased villi height (**B, C and D**, respectively). H&E staining. The scale bar represents 200 μm (magnification 100x) or 50 μm (magnification 400x). The values are expressed as the means ± SEM.

Microscopic damage was observed in the ileal samples of the irinotecan-treated WT mice (**Fig 2A–2D and Table 2**). These samples showed shortened villi with flattened and vacuolated cells, massive loss of crypt architecture, a complete disarrangement of the epithelial cell layer, and marked infiltration with inflammatory cells (**Fig 2A**). The length of the villi was decreased (**Fig 2A–2D**) compared with the intact structures observed in the saline-treated WT mice. However, irinotecan-injected MyD88- (**Fig 2A and 2B**), TLR2- (**Fig 2A and 2C**) and TLR9- (**Fig 2A and 2D**) knockout mice showed a partial and significant preservation of the height of the villi, conserved epithelial cell surfaces and less inflammatory infiltrates compared to the WT group treated with irinotecan (*P*<0.05). In addition, a semi-quantitative analysis of gut injury showed that irinotecan-injected WT mice presented a significant intestinal damage when compared to saline-administered animals, which was prevented in MyD88-, TLR2- and TLR9- knockout mice (**Table 2**).

**Table 2:** Semi-quantitative analysis of histopathological damage.

Group	Mucosal damage
	Scores
WT + saline	0(0–0)
WT + Irinotecan	2(1–3)^a^
MyD88^-/-^ + saline	0(0–0)
MyD88^-/-^ + Irinotecan	1(0–2)^b^
TLR2^-/-^ + saline	0(0–0)
TLR2^-/-^ + Irinotecan	0(0–3)^b^
TLR9^-/-^ + saline	0(0–1)
TLR9^-/-^ + Irinotecan	1(0–2)^b^

Data were analyzed using Kruskal-Wallis/Dunn’s test. The values are expressed as the median (minimum-maximum).

^a^ *P*<0.05 vs wild type control group injected with saline.

^b^ *P*<0.05 vs the wild type group injected with irinotecan. No statistical difference was found between the knockout groups that were injected either saline or irinotecan.

### Deletion of the MyD88, TLR2 or TLR9 genes prevent bacteria translocation

**Fig 3:**
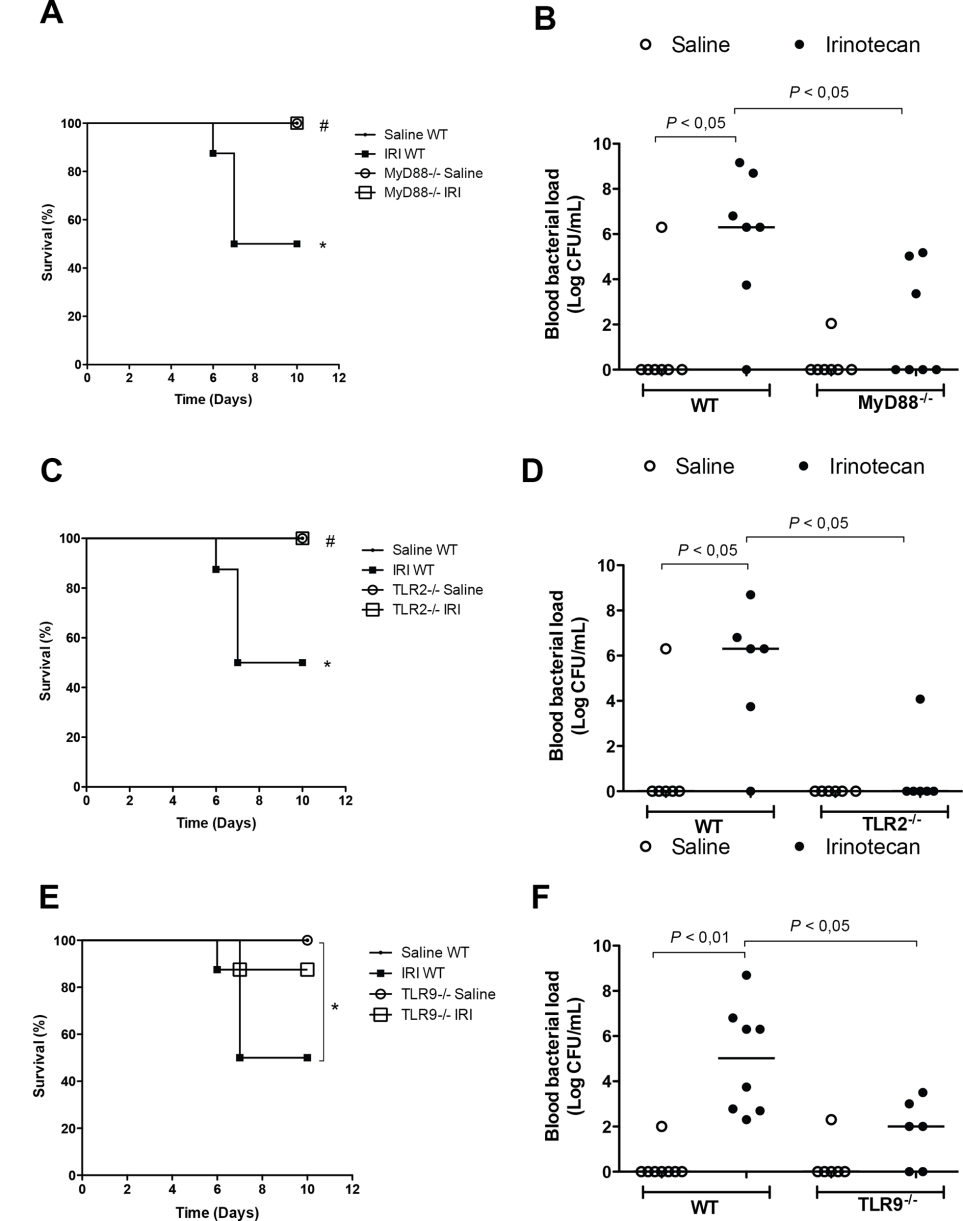
Irinotecan reduces animal survival and increases bacteremia, which were prevented in the MyD88^-/-^ and TLR2^-/-^ mice. The mice (n = 6–9) were injected for 4 days with saline (3 mL/kg) or irinotecan (75 mg/kg, i.p.) and survival was monitored during the whole experimental period. A blood sample was collected on day 7 to measure the bacterial load. The WT mice that were injected with irinotecan showed a significantly reduced survival (**A, C and E**) and increased bacteremia (**B, D and F**) versus the saline-injected group. MyD88- and TLR2-, but not TLR9-, knockout mice showed an improved survival (**A**, **C and E**, respectively). The blood bacterial load was reduced in all knockout mice (**B**, **D and F**). *P<0.05 vs the WT saline group; ^#^ *P* <0.05 knockout groups vs the WT group injected with irinotecan.

Irinotecan injection in the WT mice caused a reduced animal survival (50%) and increased bacteremia on the 7^th^ day after the first dose of irinotecan compared to saline-treated WT control group (*P*<0.05) (**Fig 3A–3F**). Additionally, animal survival was improved and bacteremia was attenuated in the MyD88-/- (100%, **Fig 3A and 3B**) and TLR2-/- (100%, **Fig 3C and 3D**) mice in comparison with the irinotecan-injected WT mice (*P*<0.05). However, deletion of the TLR9 gene prevented only the bacteremia (*P*<0.05), but not animal survival (*P* = 0.107), compared with the irinotecan-injected WT mice (**Fig 3E and 3F**).

### Irinotecan-induced diarrhea is effectively controlled in the MyD88 or TLR2 but not in the TLR9 knockout mice

As shown in **Table 3**, diarrhea was found to be statistically increased in the irinotecan-injected WT mice compared to the saline-injected WT group (*P*<0.05). In addition, the diarrheal scores of the irinotecan-injected MyD88^-/-^ and TLR2^-/-^ mice were markedly reduced (*P*<0.05) compared with the severe diarrheic events in the irinotecan-injected WT group. On the other hand, compared to their respective saline group, the irinotecan-injected TLR9^-/-^ animals showed moderate to severe diarrhea (*P*<0.05), similar to irinotecan-injected WT mice (*P*>0.05). **Table 3**also shows the significant leukopenia (*P*<0.05) in the WT and knockout animals that received irinotecan versus their respective saline-treated control groups (*P*<0.05).

**Table 3:** Diarrhea assessment and blood leukocyte counts in irinotecan-injected mice.

Group	Diarrhea assessment	Blood leukocyte count
	Scores	cells x 10^3^/μL
WT + saline	0(0–0)	3.950 ± 0.28
WT + Irinotecan	2(1–3)^a^	1.906 ± 0.2^a^
MyD88^-/-^ + saline	0(0–0)	3.625 ± 0.24
MyD88^-/-^ + Irinotecan	0(0–1)^c^	2.557 ± 0.37^b^
TLR2^-/-^ + saline	0(0–0)	3.320 ± 0.34
TLR2^-/-^ + Irinotecan	0(0–1)^c^	2.400 ± 0.17^b^
TLR9^-/-^ + saline	0(0–1)	2.580 ± 0.51
TLR9^-/-^ + Irinotecan	2(0–3)^c^	1.280 ± 0.09^b^

Parametric and non-parametric data were analyzed using ANOVA/Bonferroni’s test or Kruskal-Wallis/Dunn’s test, respectively. The values are expressed as the means ± S.E.M (parametric data) or median (minimum-maximum) for non-parametric data.

^a^ *P*<0.05 vs wild type control group injected with saline.

^b^ *P*<0.05 vs its respective knockout control group injected with saline.

^c^ *P*<0.05 vs the wild type group injected with irinotecan.

### TLR signaling is dependent on MyD88, which induces NF-κB expression

**Fig 4:**
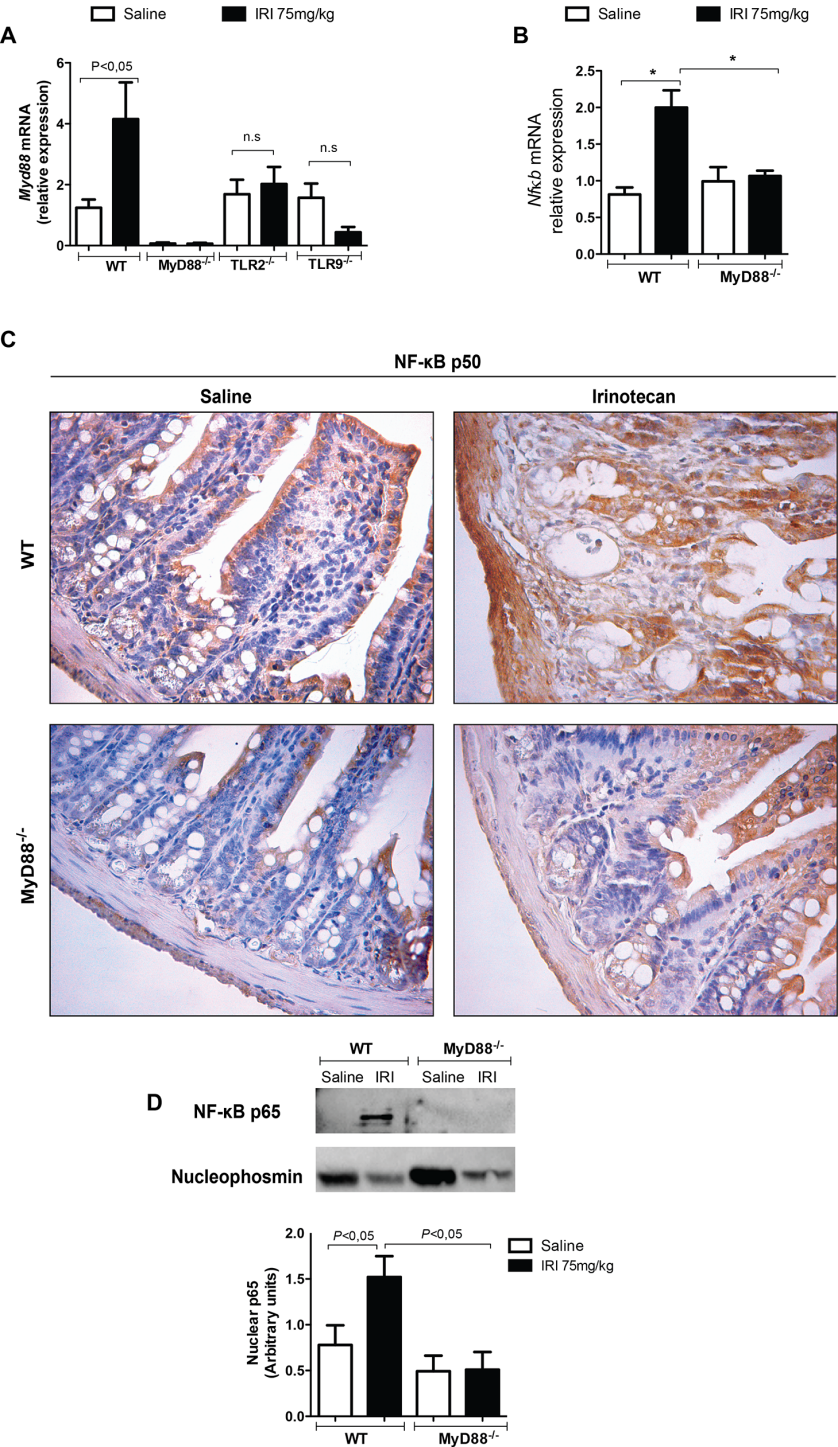
Intestinal mucositis is dependent on MyD88 and NF-κB expression. The mice (n = 6–9) were injected for 4 days with saline (3 mL/kg, open bars) or irinotecan (75 mg/kg, i.p., black bars) and were killed on the seventh day after the first dose. Ileal samples were collected and processed for MyD88 and NF-κB expression. Irinotecan injection increased MyD88 (**A**) and NF-κB (**B, C and D**) expression as detected by qPCR (**A and C**), immunohistochemistry (**B**) or western blot (**D**) versus the saline-treated WT mice. The basal expression of MyD88 was found in the irinotecan-injected MyD88-/-, TLR2-/- or TLR9-/- mice when compared to their respective saline-injected knockout controls (**A**). Deletion of the MyD88 gene also prevented the expression of NF-κB (**B, C and D**). The values are expressed as the means ± SEM.

The expression of both MyD88 (**Fig 4A**) and NF-κB (**Fig 4B, 4C and 4D**) was significantly increased in the irinotecan-injected WT mice versus the saline group (*P*<0.05). In addition, NF-κB expression is markedly increased both in cytoplasmic and nuclear (a two-fold increase) regions in irinotecan-injected WT animals (**Fig 4C and 4D**). In contrast, the expression of MyD88 (**Fig 4A**) and NF-κB (**Fig 4B, 4C and 4D**) was absent or drastically reduced in the MyD88 knockout mice. Furthermore, the expression of the adaptor protein MyD88 in TLR2-/- and TLR9-/- animals that received irinotecan remained similar to the basal levels in the saline-treated knockout mice (*P*>0.05, **Fig 4A**).

### MyD88, TLR2 and TLR9 activate a local inflammatory reaction during intestinal mucositis

**Fig 5:**
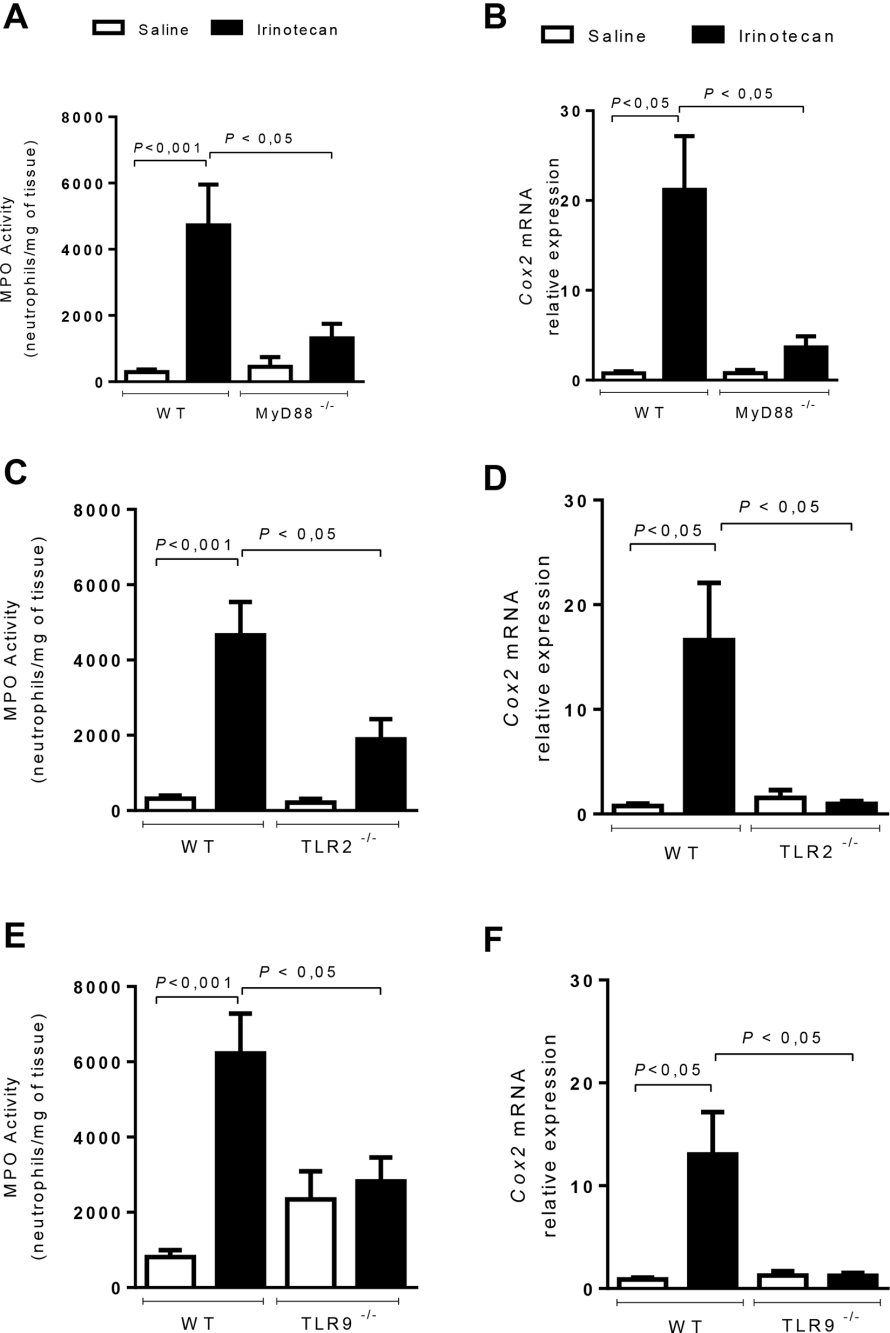
Inflammatory enzyme markers are reduced in MyD88^-/-^, TLR2^-/-^ and TLR9^-/-^ mice during intestinal mucositis. The mice (n = 6–9) were injected for 4 days with saline (3 mL/kg) or irinotecan (75 mg/kg, i.p.) and were killed on the seventh day after the first dose. Ileal samples were collected and processed for MPO activity and qPCR for COX–2. The irinotecan injection in the WT mice increased the MPO activity (**A, C** and **E**) and COX–2 expression (**B, D and F**) compared with the normal WT control animals. The MyD88-/-, TLR2-/- and TLR9-/- mice showed a significant reduction in these inflammatory enzymes compared to the irinotecan-injected WT animals. The values are expressed as the means ± SEM.

**Fig 6:**
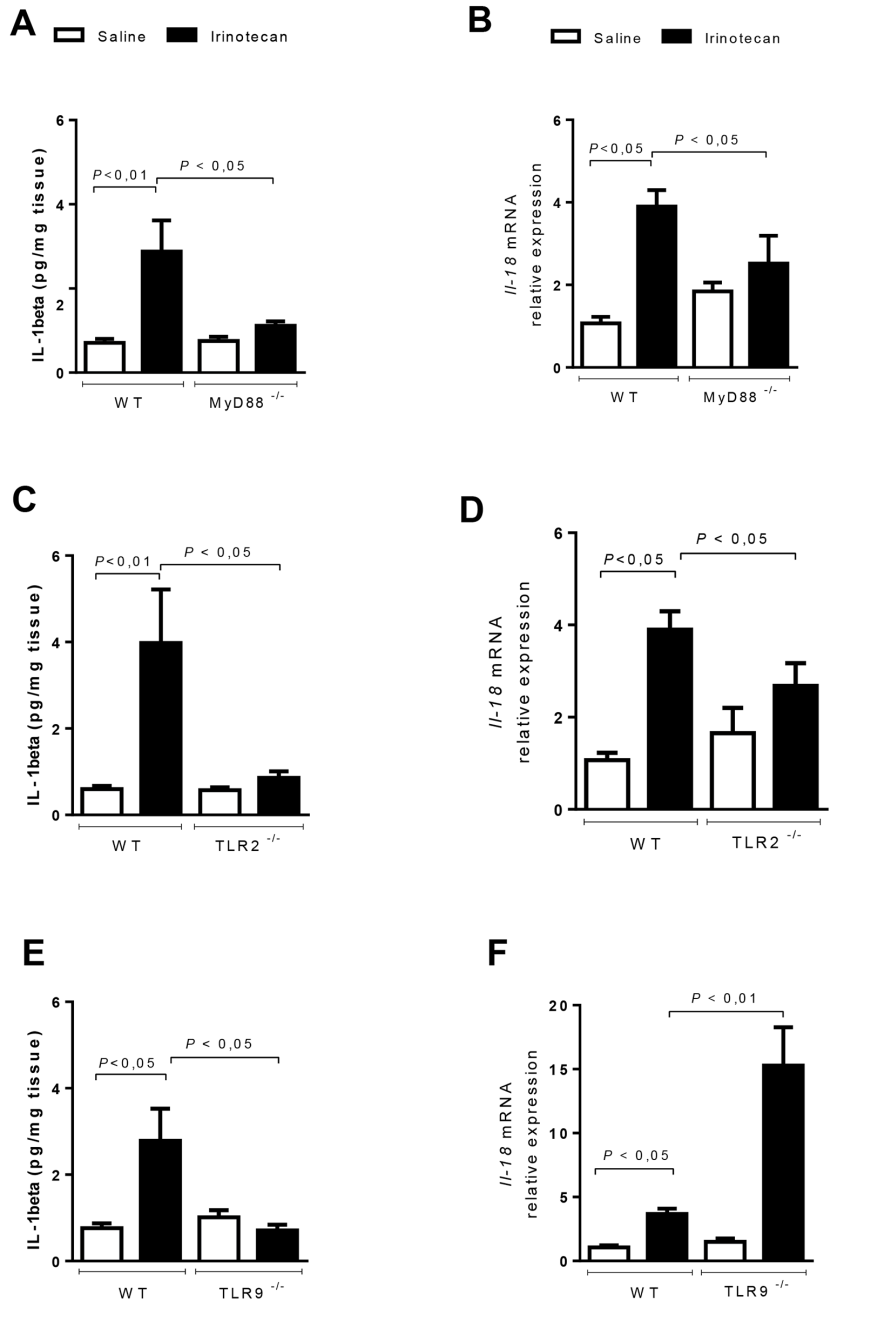
IL–1β levels and IL–18 expression are markedly decreased in the MyD88^-/-^ and TLR2^-/-^ mice. The mice (n = 6–9) were injected for 4 days with saline (3 mL/kg) or irinotecan (75 mg/kg, i.p.) and were killed on the seventh day after the first dose. Ileal samples were collected and processed for IL–1β levels (ELISA) and qPCR for IL–18. The irinotecan injection in the WT mice increased the IL–1β levels (**A, C** and **E**) and IL–18 expression (**B, D and F**) compared with the normal WT control animals. The MyD88-/- or TLR2-/- mice that received irinotecan showed a significant reduction in these inflammatory markers compared to the irinotecan-injected WT animals (**A-D**). The irinotecan-treated TLR9-/- animals showed reduced levels of IL–1β, but increased expression of IL–18 (**E and F**). The values are expressed as the means ± SEM.

The protective role of MyD88 and TLR2 and TLR9 in irinotecan-induced mucositis was associated with a pronounced reduction of the local inflammatory reaction, as detected by the measurement of myeloperoxidase activity (**Fig 5A, 5C and 5E**) and COX–2 (**Fig 5B, 5D and 5F**) and IL–1β production (**Fig 6A, 6C and 6E**). All these inflammatory markers were significantly increased in irinotecan-administered WT animals (**Figs 5 and 6, Panels A-F**). Of note, the expression of IL–18 induced by irinotecan (**Fig 6C and 6D**) was reduced in the TLR-2-/- and MyD88-/-, but not in the TLR9-/-, mice (**Fig 6F**), in which the expression was markedly enhanced compared with irinotecan-injected WT mice.

**Fig 7:**
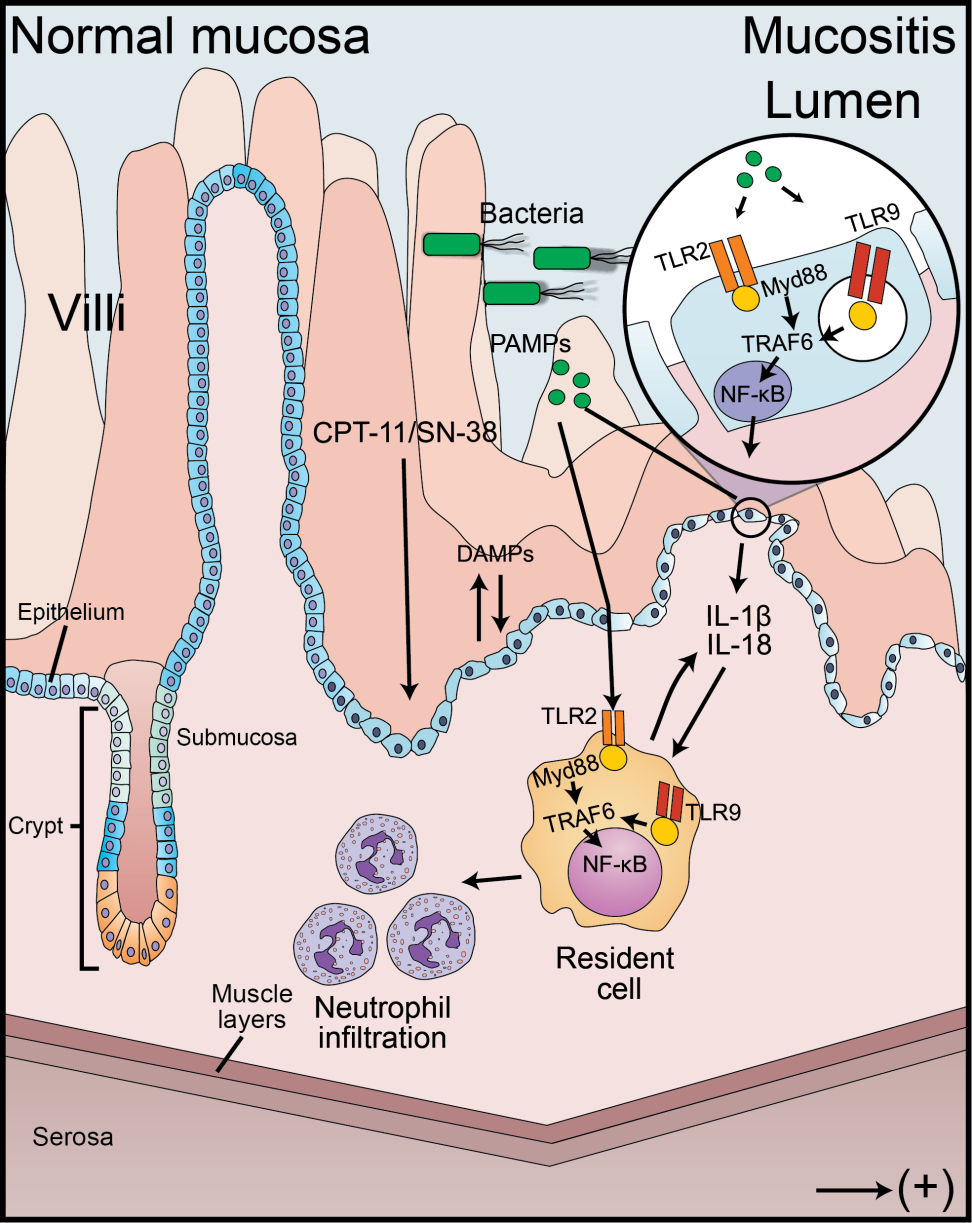
Hypothesis for the development of intestinal mucositis. Irinotecan (CPT–11) is metabolized in the liver into the active compound SN–38, which in turn is inactivated through glucuronidation to SN-38G. SN-38G is eliminated through the common bile duct into the intestinal tract where it is re-activated by Gram-negative bacteria containing beta-glucuronidase enzymes. In the intestinal lumen, the active SN–38 leads to epithelial damage, allowing the enteric bacteria to translocate. Pathogen-associated molecular patterns (PAMPs) and Damage-associated molecular patterns (DAMPs) are recognized by the toll-like receptors, which signal through the MyD88 adaptor protein and TNF receptor-associated factor 6 (TRAF6) to activate NF-κB and cytokine synthesis. This process contributes to neutrophil recruitment to the site of infection, amplifying the damage.

Hypothesis model of the inflammatory cascade activated during the pathogenesis of irinotecan-induced intestinal mucositis is shown in **Fig 7**. Pathogen and damage associated molecular patterns (PAMPs and DAMPs) trigger the synthesis of pro-inflammatory cytokines (e.g. IL–1β and IL–18) and enzymes (COX–2, for instance) through the activation of TLR2 and TLR9 signaling pathway. These receptors signal through the MyD88 adaptor molecule and TNF receptor-associated factor 6 (TRAF6) to activate NF-κB transcription function. The recruitment of inflammatory cells, such as neutrophils, leads to the amplification of intestinal injury and the development of mucositis.

## Discussion

In the present study, we showed that the adaptor protein MyD88 has a major detrimental contribution to the pathogenesis of intestinal mucositis and is possibly activated by toll-like receptors. The genetic deletion of MyD88, TLR2 or TLR9 prevented the progression of the irinotecan-related intestinal mucositis as detected by the attenuation of several parameters including tissue damage, neutrophil infiltration and expression of inflammatory mediators. As a consequence of the reduced mucositis, the blood bacterial load and an improvement in the survival of the animals were observed.

Interestingly, we found that the expression of TLR9, MyD88 and TRAF6 were significantly increased following irinotecan injection in wild type mice. However, TLR2 expression did not change. One possibility that could explain such apparent conflicting result in regard to TLR2 is that the mRNA translation into protein in WT mice might be highly activated in irinotecan group, but not in saline-injected animals. In spite of the basal expression of TLR2, genetic deletion to this receptor broadly prevented the development of intestinal mucositis, suggesting the important role of TLR2 in the pathogenesis of intestinal mucositis.

Irinotecan-induced intestinal damage is a well-described phenomenon in the literature [**5,14,15,24,35**]. Those studies showed that irinotecan induces a marked loss of the epithelial cell lining, shortening of the villi, expression of inflammatory mediators and infiltration of leukocytes into the lamina propria in a manner similar to that found in the present research. In addition, Nakao and colleagues [36] suggested that irinotecan damages claudin–1 and occludin, leading to disorders in the intestinal epithelial barrier. This damage allows bacterial translocation, which may contribute to an enhancement of the mucositis. Bacterial translocation can be stratified to three levels: a) local- level I- (mesenteric lymph nodes), b) regional- level II (portal blood and /or liver), and c) systemic- level III (peripheral blood, spleen). In the present study, we observed a systemic level III bacterial translocation in the irinotecan-injected WT mice, an effect that correlated with the severity of the mucositis and was not observed in TLR2 and TLR9 knockout mice. We also observed a significant regional level II bacterial translocation to the liver in irinotecan-injected wild type mice when compared with saline-treated group. However, no statistical difference (P>0.05) was observed between wild type mice and knockout animals that were injected with irinotecan (data not shown). These results might indicate that the systemic bacterial translocation is delayed in the lack of TLRs signaling, which reinforces the deleterious role of bacterial translocation as a crucial event in the development of irinotecan-associated mucositis.

The central roles of TLR2 and TLR9 in the development of intestinal mucositis induced by another anticancer agent, doxorubicin, have also been described in a previous study [**21**]. Furthermore, the literature describes the involvement of TLRs in other gastrointestinal inflammatory diseases. In clinical inflammatory bowel diseases, for instance, activation of TLRs appears to have a role in the development of damage to the large and small intestines of patients because these receptors are found to be markedly expressed in the intestinal epithelium of those patients [**37,38**]. Furthermore, TLRs have been suggested to play a critical role in spontaneous, commensal-dependent experimental colitis [**9**]. In contrast do the detrimental role of TLRs during doxorubicin-induced mucositis, the activation of these receptors seems to be protective in the mucositis induced by another anticancer agent, methotrexate [**19, 20**]. These findings clearly indicate that the signaling pathways involved in intestinal damage during mucositis vary according to the drug, which might suggest the use of specific therapeutic approaches to prevent these toxicities.

Since we have previously shown the deleterious role of IL–18 in the pathogenesis of irinotecan-induced intestinal mucositis [**15**] and considering that MyD88 is the key signaling molecule downstream of several TLRs and members of the interleukin–1 receptor superfamily [**39**], we investigated whether this adaptor protein is involved in the pathogenesis of mucositis. In fact, the expression of MyD88 was markedly increased in the irinotecan-injected wild-type mice, but not in the TLR2- or TLR9-knockout animals. In addition, similar to the TLR2^-/-^ and TLR9^-/-^ mice, the mice with a genetic deletion of MyD88 were markedly protected from the development of mucositis, as indicated by a reduced production of inflammatory markers and intestinal damage. Consequently, they also demonstrated a reduced bacteremia. These results might suggest that the TLRs, and possibly IL1-family of cytokines, signal through MyD88 causing the irinotecan-related intestinal damage.

It is well known that the TLR/MyD88 signaling pathway induces nuclear factor kappa B (NF-κB) activation [**40**]. Moreover, there is evidence that the expression of NF-κB is increased during mucositis [**41**]. Here, we determined that the MyD88 knockout mice exhibited a reduced NF-κB activation, as well as the expression of pro-inflammatory mediator in the intestinal samples. These results clearly suggest that the transcription of this factor with consequent transcription of inflammatory mediators is downstream of TLR/MyD88 pathway. Similarly, it was recently shown that TLR2 activation in BV–2 microglia cells leads to the induction of the MyD88/PI3-kinase/AKT/NF-κB signaling pathway, which mediates the expression of several cytokines as well as COX–2 and iNOS [**42**]. Possibly the striking protective response shown in regard to the MyD88 protein reflects not only the modulation of signaling by the toll-like receptors, but also the broader modulation of members of the IL–1 family. In a previous study by our group, we found that irinotecan-induced intestinal damage might be inhibited by the administration of pentoxifylline, which inhibits the production of IL–1 as well as other cytokines [**14**].

The data discussed above indicate that the inflammatory parameters and signs of intestinal damage were prevented in the TLR2-, TLR9- and MyD88-knockout animals. However, with respect to the diarrhea and survival rate parameters, the TLR2-/- or MyD88-/- mice showed diminished diarrheic events and improved survival in comparison with the WT mice, whereas the TLR9-knockout mice were not protected. The late diarrhea due to irinotecan is known to be mediated by eicosanoids [**43,44**] and IL–18 [**15**]. In our study, genetic deletion of TLR9 or MyD88 led to a lower expression of COX–2, which argues against the participation of eicosanoids in the diarrhea that remained in the irinotecan-injected TLR9^-/-^ mice. On the other hand, the expression of IL–18 remained significantly high in those mice. The literature reports that, although IL–1β and IL–18 belong to the same cytokine family, they have different processing mechanisms [**45**]. The transcription of pro-IL–1β is mediated by the activation of the NF-κB, whereas pro-IL–18 is constitutively expressed in the majority of cell types. However, the production of both cytokines is dependent on the same mechanism, i.e., activation of caspase–1 and 11 [**45**]. Since irinotecan is known to activate the NLRP3 inflammasome in a reactive oxygen species-dependent manner [**46**], IL–18 processing might occur even in the TLR9^-/-^ mice. In contrast, similar to the results for TLR2 or MyD88, the lack of TLR9 signaling would reduce MyD88/NF-κB-dependent expression of pro-IL–1β with the consequence that IL–1 production would be reduced. Due to the persistent diarrhea and IL–18 production in the TLR9^-/-^ animals, their survival was reduced.

It is worth mentioning that the deletion of the TLR2, TLR9 or MyD88 genes did not seem to affect the irinotecan-induced cytotoxic effects because the leukopenia was still observed [**5**]. Furthermore, the protective effect against the development of the inflammatory reactions and diarrhea observed in the MyD88 and TLR2 knockout animals contributed to a significantly better clinical condition and an improved animal survival. Therefore, the stimulation of the immune response by the intestinal microbiota via TLR/MyD88 and the activation of IL–1 family of cytokines, which also signal through MyD88 adaptor protein, seem to be relevant in the context of the mucositis associated with this anticancer treatment. This pathway orchestrates the activation of NF-κB with the consequent production of cytokines and other inflammatory mediators (**Fig 7**). To the best of our knowledge, this is the first time that the TLR/MyD88/NF-κB pathway has been implicated in the mechanisms of damage involved in irinotecan-related intestinal mucositis. The pharmacological modulation of these target receptors might have a clinically relevant therapeutic impact.
